# Enhancing the Mechanical Properties of Regenerated Cellulose through High-Temperature Pre-Gelation

**DOI:** 10.3390/ma17194886

**Published:** 2024-10-05

**Authors:** Yuxiu Yu, Weiku Wang, Yaodong Liu

**Affiliations:** 1 CAS Key Laboratory of Carbon Materials, Institute of Coal Chemistry, Chinese Academy of Sciences, 27 Taoyuan South Road, Taiyuan 030001, China; yuyuxiu@sxicc.ac.cn (Y.Y.); weikuu7039@163.com (W.W.); 2 Center of Materials Science and Optoelectronics Engineering, University of Chinese Academy of Sciences, Beijing 100049, China; 3 University of Chinese Academy of Sciences, 19 Yuquan Road, Beijing 100049, China

**Keywords:** cellulose, pre-gelation, hydrogel, dry-jet wet spinning, mechanical properties

## Abstract

This paper investigates the effects of pre-gelation on cellulose dissolved in LiCl/DMAc solutions to enhance the properties of regenerated cellulose materials. This study focuses on characterizing the crystallinity, molecular orientation, and mechanical performance of cellulose fibers and hydrogels prepared with and without pre-gelation treatment. X-ray diffraction (XRD) analysis reveals that crystallinity improvement from 55% in untreated fibers to 59% in fibers pre-gelled for 3 and 7 days, indicating a more ordered arrangement of cellulose chains post-regeneration. Additionally, XRD patterns show improved chain alignment in pre-gelled fibers, as indicated by reduced full width at half the maximum of Azimuthal scans. Mechanical testing demonstrates a 30% increase in tensile strength and a doubling of the compression modulus for pre-gelled fibers compared to untreated fibers. These findings underscore the role of pre-gelation in optimizing cellulose material properties for applications ranging from advanced textiles to biomaterials and sustainable packaging. Future research directions include further exploration of the structural and functional benefits of pre-gelation in cellulose processing and its broader implications in material science and engineering.

## 1. Introduction

Cellulose is one of nature’s most abundant natural polymers, renowned for its good mechanical properties, bio-degradability, bio-compatibility, and bio-safety [1]. Cellulose has gained significant attention from researchers due to its unique properties. Cellulose has a very complex aggregate structure, hydrogen bonds, and van der Waals force [2]. Since cellulose degrades before melting upon heating, the processes of dissolution and regeneration are pivotal for converting native cellulose into various forms such as fibers, films, hydrogels, and aerogels [3,4]. Commonly employed solvents for cellulose include ammonia/NH4SCN [5], NaOH/(thio)urea [6,7,8], LiCl/dimethylacetamide (DMAc) [9,10], N-methylmorpholine-N-oxide/water [11], and ionic liquids [12,13]. Among these options, the LiCl/DMAc system offers several advantages: it can dissolve high molecular weight cellulose (M_w_ > 10^6^ g/mol) [14], undergoes much milder dissolution conditions and minimal chemical degradation, and exhibits excellent solution stability. As a result, LiCl/DMAc finds widespread application in various scenarios, including analysis, processing, and chemical modifications.

The dissolution status of cellulose chains in the solution and the subsequent phase separation behaviors greatly affect the structures and performances of the regenerated cellulose. In certain solvent systems, such as the NaOH/urea [15,16] system and LiCl/DMAc [17,18], the irreversible gelation of cellulose could occur during heating or cooling. This phenomenon underscores the complexity of cellulose dissolution and regeneration processes and emphasizes the need for precise control over experimental conditions to achieve the desired structures and properties. In a study by Sameer et al., cellulose dissolved in 2-ethyl-3-methylimidazolium phosphate at 25 °C and 80 °C exhibited a liquid crystal phase at the lower temperature [19]. Notably, the tensile modulus of regenerated cellulose fibers from the 25 °C solution was 40% higher than those from the 80 °C solution, indicating a significant impact of the dissolution status on mechanical properties. Our prior observations revealed that cellulose dissolved in LiCl/DMAc experiences irreversible gelation upon heating, likely due to temperature-dependent chain interactions [17]. Further investigation into the influence of pre-gelation on regenerated cellulose could offer valuable insights into optimizing its properties for various applications.

While there is extensive research on the fabrication of high-performance fibers, such as polyacrylonitrile fibers and polyethylene fiber [20,21,22], utilizing either pre-gelation or post-gelation methods, there has been relatively less focus on the preparation of regenerated cellulose fibers through gel methods. Compared with wet-spun fibers, the stretchability of gel-spun fibers is commonly greatly improved, and the resulting fibers could exhibit both high orientation and strength. In this study, cellulose was dissolved in LiCl/DMAc, and the solutions were subjected to either high- or low-temperature pre-treatments. Notably, irreversible pre-gelation of the cellulose solution occurred under high-temperature treatment. Regenerated cellulose fibers offer significant potential for various applications owing to their renewable nature, biodegradability, and exceptional mechanical properties. Utilizing gel methods for their preparation presents unique advantages, including precise control over fiber morphology and mechanical characteristics. By addressing this research gap in the existing literature, new opportunities could emerge for producing regenerated cellulose fibers and hydrogels with significantly enhanced properties. This could stimulate further exploration and innovation in the field, leading to advancements in various applications ranging from textiles to biomedical engineering.

## 2. Materials and Characterization

**Materials:** Dehydrated lithium chloride (LiCl, >99.0%) and DMAc (>99.0%) were purchased from E-Aladdin (Shanghai, China). Deionized water was prepared using an ultra-pure water system (model ROP-15L, >15 MΩ) made by Heal Force Bio-Meditech Holdings Ltd. Corp (Shanghai, China). Microcrystalline cellulose (MCC, cotton-based) was sourced from Henan Hengrui Food Additive Ltd. Corp (Zhengzhou, China).

**Cellulose activation:** MCC powders were vacuum dried at 60 °C until they reached a constant weight. Then, 40 g of MCC powders were soaked in 400 mL deionized water at 30 °C for 24 h, vacuum filtered, and then vacuum dried at 60 °C until no further weight loss was observed. Finally, the water-treated MCC was then subjected to treatment with DMAc under the same conditions as the water treatment.

**Solution preparation:** Then, 8 g of LiCl was mixed with 92 g of DMAc at 60 °C under continuous magnetic stirring for 24 h. Then, 10 g pre-treated MCC was slowly added into 90 g LiCl/DMAc solvent in a necked glass bottle, and was magnetically stirred at 110 °C for 50 min. Finally, the solvent was transferred inside a double-layer reactor set at 5 °C and mechanically stirred for 12 h.

**Pre-gelation:** The prepared MCC solution was poured into bottles, which were then filled with nitrogen to create an inert atmosphere. The bottles were sealed and placed in an oven at 60 °C for different durations: 0 days (G0), 3 days (G3), and 7 days (G7).

**Regenerated cellulose hydrogel:** The MCC solution was shaped by a centrifugal tube, and immersed in water for 48 h. For SEM measurements, the regenerated MCC hydrogel was solvent exchange by tert-butyl alcohol, and then dried inside a freeze-dryer (model VFD2000, made by Bio-cool Lab Instrument Co., Beijing, China); the dried hydrogels were used for SEM characterizations.

**Regenerated cellulose fiber:** The MCC solution was transferred into a stainless-steel syringe; the gas bubbles were removed at room temperature for 24 h under vacuum. Fiber spinning was performed using laboratory self-design fiber-spinning equipment, which consists of a vertical screw extruder, a water bath, drawing rolls, and a winding unit. The spinneret is a single-hole spinneret with a hole diameter of 200 microns and an L/D ratio of 3. The extrusion velocity and winding velocity were set to be 1 m/min and 10 m/min, respectively. The water bath was about 2 m long and maintained at 5 °C. After spinning, the fiber was immersed in water for 48 h to remove the residual solvent. Finally, the fiber was dried at 60 °C under vacuum.

**Characterizations:** The surface morphology was examined using a field emission scanning electron microscope (FE-SEM, Model JSM-7001F, JEOL Ltd., Tokyo, Japan) after gold sputtering for enhanced conductivity. The cross-sectional views of the fibers were observed using a polarizing microscope (Olympus CX31-P, Tokyo, Japan) following resin embedding and sectioning with a microtome to achieve 0.02 mm thick slices. The dissolution behavior of MCC in DMAc/LiCl solvent was examined using a polarized optical microscope. The mechanical properties of the hydrogels were evaluated using a Universal Tensile Tester. Prior to testing, each hydrogel specimen was prepared by cutting it into a cylindrical shape with a diameter of 10 mm and a length of 8 mm. Compression testing was conducted at a rate of 2 mm/min. The compressive modulus (*E*) was determined by calculating the slope within the strain range of 0.05 to 0.1. The tensile properties of the fibers were tested according to GB/T14337-2008 standard [23]. The reported values are averaged from at least 20 independent tests and the error bars are standard deviations. Rheological characterization of MCC solutions was performed using a rotational rheometer (MCR-302, Anton-Paar GmbH, Graz, Austria) equipped with 2 mm diameter parallel plates set at a 1 mm gap distance. Dynamic amplitude sweeping involved increasing strain amplitudes from 0.0001 to 100 at a fixed frequency of 1 rad/s and at a temperature of 20 °C. Additionally, thermal gelation behavior was studied using a dynamic strain amplitude of 0.0025 and a constant frequency of 1 rad/s, while heating or cooling the sample at a rate of 1 °C/min. The crystal structure of the regenerated cellulose fiber was analyzed using X-ray diffraction (XRD). The crystallinity index (CI) was calculated by peak fitting method (Figure S1 in ESI). The degree of orientation (*f*) of cellulose crystals within fibers can be characterized using the Herman’s formula, which compares the alignment of the molecular chain axis relative to the fiber axis [24]. The formula is expressed as follows:

$$f=\frac{3<{cos}^{2}\theta >-1}{2}$$

$$<{cos}^{2}\;\theta >=\frac{\int_{0}^{\pi }I\left(\theta \right)\cos^{2}\theta d\theta }{\int_{0}^{\pi }I\left(\theta \right)d\theta }$$

where *θ* is the angle between the direction of the cellulose crystal and the fiber axis, ⟨⋅⟩ denotes the average over all crystals. Small-angle X-ray scattering (SAXS) measurements were conducted using an X-ray diffractometer (model DX-2700, Fangyuan instrument, Dandong, China) equipped with Cu K_α_ irradiation source (λ = 0.15418 nm, 40 kV, 30 mA). The experimental setup involved scanning with a step size of 0.01° over the angular range from 0.5° to 4°, and each data point was collected with a signal collection duration of 2 s.

## 3. Results

### 3.1. Effect of Pre-Gelation on MCC Solution

**Figure 1:**
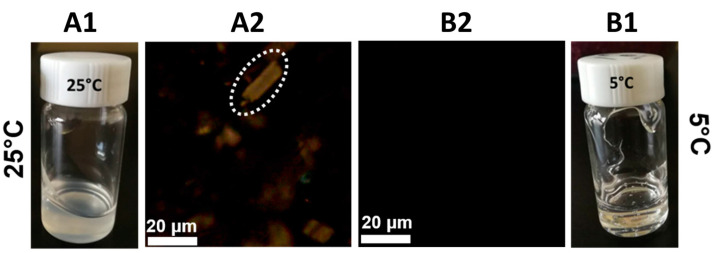
(**A1**,**B1**) Photograph, (**A2**,**B2**) polarization optical images of 10 wt% cellulose/LiCl/DMAc solution dissolved at different temperatures. The white dashed circle contains insoluble cellulose particles.

Solution rheology is essential in fiber spinning as it directly impacts the spinnability, fiber uniformity, and overall quality of the final product. The rheological behavior of a polymer solution is largely determined by the conformation and interactions of the polymer chains within the solvent, which are in turn influenced by the dissolution process and thermal history. At a dissolution temperature of 25 °C, the MCC solution appeared turbid and incomplete dissolution was observed, as depicted in Figure 1 While cellulose particles were not visible under regular optical microscopy, polarized light revealed the presence of insoluble cellulose particles dispersed within the solution. In contrast, when MCC was dissolved at 5 °C, the solution achieved complete dissolution and exhibited transparency. The WAXD patterns also suggest undissolved cellulose at a higher temperature (Figure S2 in ESI), and frequency-dependent rheological data indicate that lower dissolution temperature improves the homogeneity of the cellulose solution (Figure S3 in ESI). Typically, lower temperatures slow down the dissolution kinetics, allowing more time for the solvent to penetrate and interact with the cellulose structure. However, this observation suggests that higher concentrations of cellulose can be more effectively dissolved at lower temperatures within the DMAc/LiCl system. While DMAc and LiCl form a solvent system that is known to dissolve cellulose effectively, at lower temperatures (5 °C), the solvent and ion system may interact more favorably with cellulose chains, enhancing their solubility and leading to complete dissolution. However, at higher temperatures (25 °C), the interactions may weaken or become less effective, resulting in incomplete dissolution and the presence of insoluble cellulose particles.

**Figure 2:**
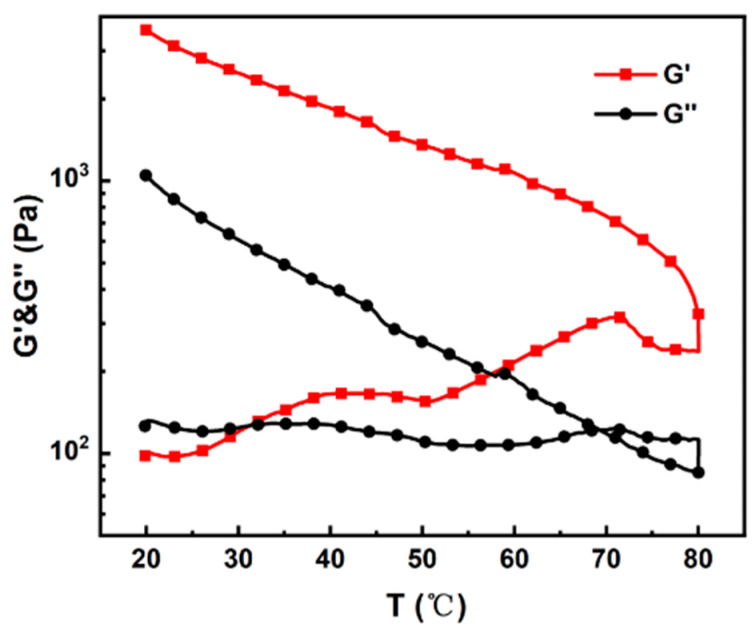
Temperature-dependent storage and loss moduli of MCC solution. Temperature was raised from 20 to 80 °C, and then decreased to 20 °C at a ramping rate of 1 °C/min.

In previous research [25,26], we observed that irreversible gelation of the cellulose solutions occurs at high temperatures within the DMAc/LiCl system. Figure 2 illustrates that gelation initiates at a cellulose concentration of 10 wt% and a temperature of 31 °C, leading to the conversion of the solution into a gel state. At a temperature higher than 70 °C, cellulose chains may degrade, leading to a decrease in dynamic moduli. This irreversible transformation indicates a critical point where the solubility of cellulose in the DMAc/LiCl solvent system is compromised.

**Figure 3:**
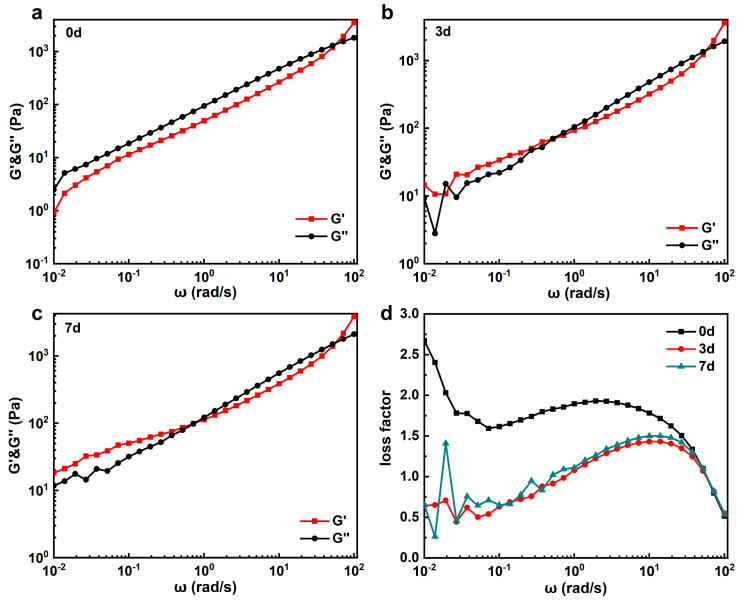
Angular frequency-dependent rheological data of MCC solutions underwent different pre-gelation durations. (**a**–**c**) Angular frequency-dependent rheological data at 0d, 3d, and 7d, respectively. (**d**) Angular frequency-loss factor curves at different pre-gel times.

Without pre-gelation treatment, Figure 3a illustrates that cellulose solutions exhibit viscous characteristics in the low-frequency region. This behavior suggests a predominantly liquid-like state where the cellulose chains exhibit viscous flow under shear stress. At a frequency higher than 80 rad/s, the solution became more elastic. Following pre-gelation at 60 °C for 3 and 7 days, as shown in Figure 3b,c, respectively, frequency scanning of the cellulose solutions reveals a transition to elastic behavior even in the low-frequency region. This change indicates the formation of a gel-like structure characterized by polymer entanglements and network formation. The transition from viscous to elastic behavior signifies a shift towards a more solid-like state, where the MCC molecules are interconnected within a gel network. Moreover, as the frequency increases during the frequency sweep, disturbances disrupt the gel network, leading to an increase in the loss factor (Figure 3d). The increase in the loss factor reflects a decrease in the elastic properties and a shift back towards a more fluidic state. Such behavior indicates the dynamic nature of the gel formed in the MCC solutions, where mechanical disturbances, such as shearing during fiber extrusion, might temporarily disrupt the gel network and alter its rheological properties. The instability of the LiCl/DMAc solution at high temperatures and the complex interplay between polymer entanglements and external mechanical forces in MCC gelation processes further complicate the processing of regenerated cellulose, indicating both the temperature and shear sensitivity of the DMAc/LiCl solvent system.

**Figure 4:**
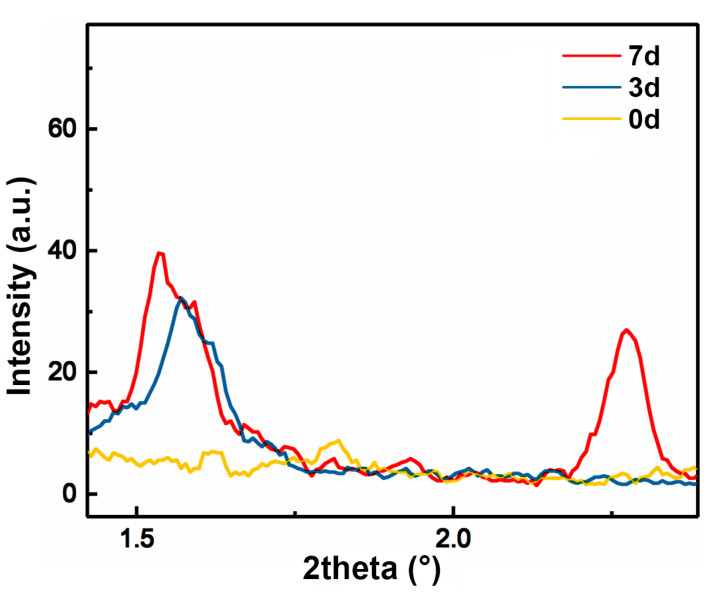
SAXS profiles of MCC solution with different pre-gelation durations.

To further investigate the gelation behavior of cellulose, SAXS was employed to analyze cellulose solutions before and after pre-gelation for 3 days and 7 days, and the corresponding solvent-subtracted scattering profiles are plotted in Figure 4. Initially, the MCC solution without pre-gelation treatment shows no visible diffraction peak, indicating a lack of ordered structures in the cellulose solution. However, after 3 days of pre-gelation treatment at 60 °C, a scattering peak at 2 *θ* = 1.6° emerges in the SAXS pattern. This peak suggests the formation of some long-range (*d*~5.5 nm) ordered structures within the cellulose solution. This peak became more pronounced after 7 days of pre-gelation, and an additional peak at 2 *θ* = 2.3° (*d*~3.8 nm) was observed, suggesting further structural organization and aggregation of cellulose chains. Those structural changes suggest that over time, the cellulose chains in the solution might assemble together and organize into ordered aggregates during pre-gelation at 60 °C. These ordered structures are likely due to increased chain interactions and network formation among cellulose chains, which are facilitated by the pre-gelation process. A longer pre-gelation time leads to more aggregation and ordering of cellulose chains. These structural changes could potentially lead to improved mechanical properties and orientation in the resulting regenerated cellulose.

### 3.2. Effect of Pre-Gelation on Regenerated Hydrogel

**Figure 5:**
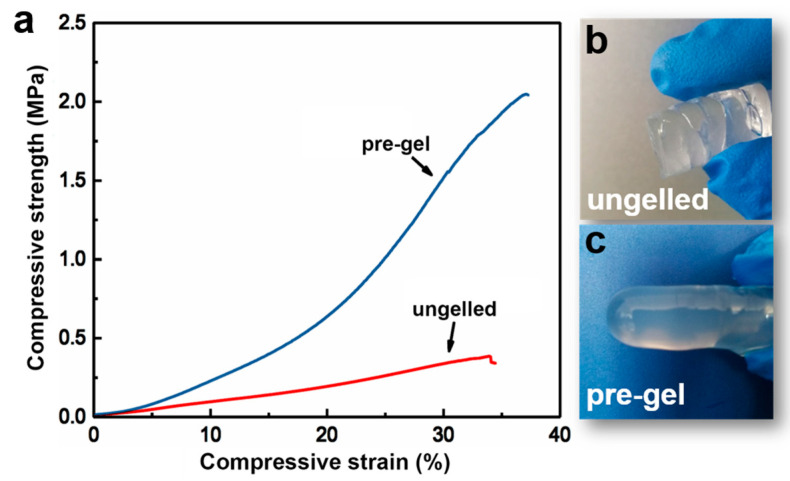
(**a**) Stress–strain curves of cellulose hydrogels. (**b**,**c**) Photographs of ungelled cellulose hydrogel and pre-gel cellulose hydrogel, respectively.

In order to further understand how the irreversible gelation of cellulose solutions affects the structures and properties of regenerated cellulose, cellulose hydrogels were prepared using a solvent exchange method. By comparing the properties of hydrogels formed with and without pre-gelation treatment, insights into the impact of gelation on the mechanical properties, structural integrity, and overall performance of the regenerated cellulose can be gained. The typical tensile compressive–strain curves for pre-gelation and non-gelation cellulose hydrogels are shown in Figure 5. The data indicate that the pre-gelation hydrogel exhibits significantly superior mechanical properties compared to the hydrogel without pre-gelation treatment. Specifically, the compression modulus of the hydrogel after pre-gelation at 60 °C for 7 days is 2.9 MPa, which is twice as high as that of the prepared hydrogel without undergoing pre-gelation. Additionally, the compressive strength of the pre-gelation hydrogel approaches 2.3 MPa, which is 350% higher than that of the untreated hydrogel. The formation of cellulose chain aggregation network during pre-gelation leads to significant enhancements in mechanical properties, which suggests the effectiveness of the pre-gelation process in reinforcing the structural integrity and durability of the resultant cellulose hydrogel, making it more suitable for applications requiring robust mechanical performance.

**Figure 6:**
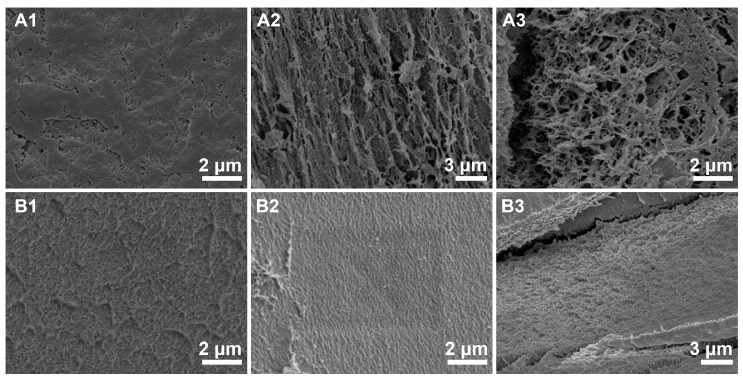
SEM images of regenerated cellulose from hydrogel (**A1**, **A2**, and **A3**) Surface, cross-section, and fracture surface of without pre-gelation, respectively. (**B1**, **B2**, and **B3**) Surface, cross-section, and fracture surface of after pre-gelation for 7 days.

To better elucidate the relationship between the structure and properties of cellulose hydrogels, SEM was employed to examine the internal structure of these materials. To effectively preserve the internal structure of the hydrogels, water was solvent-exchanged with tert-butanol before freeze-drying. The SEM images in Figure 6 reveal significant structural differences for cellulose materials regenerated from different pre-gelation conditions. Unlike the regenerated cellulose hydrogel from cellulose/NaOH+urea solutions [27,28], those prepared from cellulose/DMAc+LiCl solutions exhibit a more compact structure with smaller pores (Figure 6A1–A3). The SEM images of the hydrogels without pre-gelation treatment are characterized by numerous pores ranging from 100 nm to 500 nm. We also observed that other regenerated cellulose contained lots of fibrils and sheets with a thickness of tens of nanometers. In contrast, the SEM images of the hydrogels with pre-gelation treatment show a smooth surface and a cross-section with no large pores. The surface is very flat, and even the fracture defects do not display any significant pores with a G larger than 200 nm. This compact structure is likely due to the cellulose chain aggregation network formed during the pre-gelation process, which enhances the material’s density and mechanical properties.

A possible explanation for the formation mechanism of the hydrogel involves the distinct behaviors of cellulose chains in solution with and without pre-gelation treatment. In solutions without pre-gelation, cellulose chains are randomly dispersed and less structured. Cellulose microfibrils regenerate during the solvent exchange process with water, resulting in a network of loosely connected fibrils. This loose network allows for more significant shrinkage during drying due to the lack of structural integrity, leading to the formation of larger micropores and irregular fiber accumulation. This observation aligns with findings reported by Miri et al., who demonstrated that cellulose hydrogels without any pre-treatment exhibited larger pore sizes and non-uniform structures due to the unrestricted fibril movement and aggregation during regeneration [29]. On the other hand, for the solution after pre-gelation, cellulose chains have already begun to form a preliminary network due to the pre-gelation process. This pre-formed network provides a template that promotes further cross-linking and alignment of cellulose molecules during the water exchange process. As a result, the regenerated cellulose forms a denser and more uniform 3D network with much smaller pores. This is consistent with the results observed by Wu et al., who found that pre-gelation treatment led to the formation of a more compact hydrogel structure, characterized by reduced pore size and increased mechanical strength [30]. The pre-gelation treatment reduces the extent of shrinkage during drying by maintaining a more robust structure, leading to fewer micropores and a smoother surface. This denser network structure formed during pre-gelation is better able to resist the forces during drying, minimizing shrinkage and producing a more uniform and smoother hydrogel compared to those prepared without pre-gelation treatment.

### 3.3. Effect of Pre-Gelation on Regenerated Fibers

**Figure 7:**
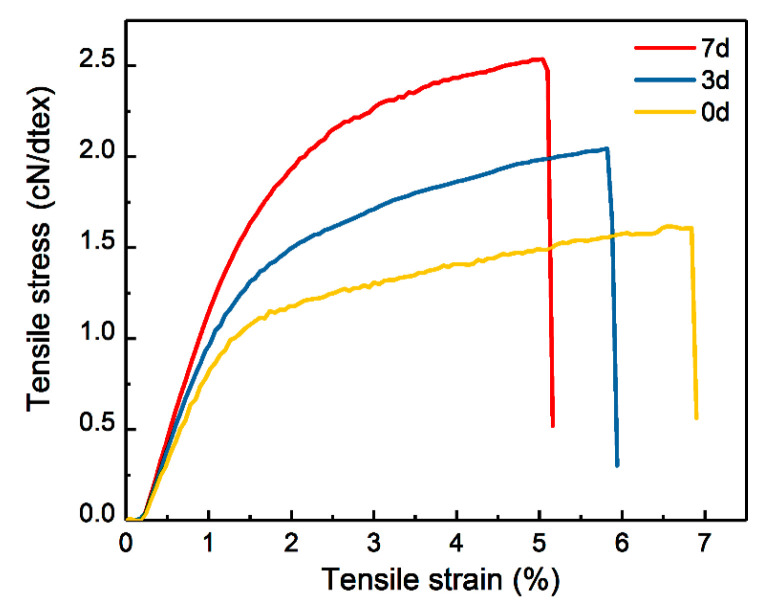
Typical stress–strain curves of regenerated cellulose fibers.

The cellulose solutions with and without pre-gelation were spun into fibers. The typical tensile stress–strain curves of G0, G3, and G7 cellulose fibers are shown in Figure 7. The detailed data are summarized in Table S1 in ESI. As the duration of pre-gelation of the cellulose solution increases, both the Young’s modulus and tensile strength of the regenerated cellulose fibers significantly improve, although the breaking strain decreases slightly. Specifically, the mechanical properties of the fibers increase by 30% after 3 days of pre-gelation but only see an additional 10% improvement from 4 to 7 days. Notably, when the gelation time extends to 14 days, the spinnability of the cellulose solution diminishes.

**Figure 8:**
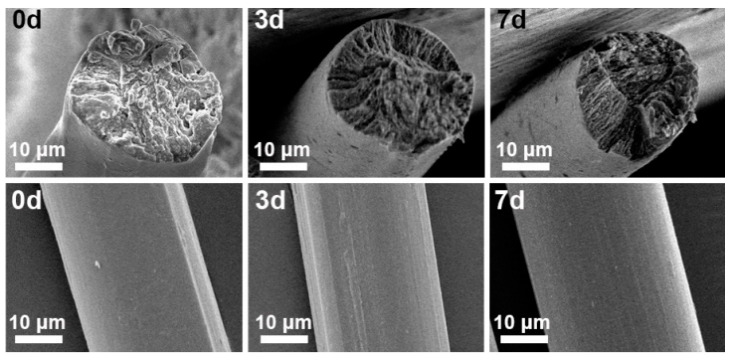
Cross-section (**upper**) and surface morphology (**under**) of regenerated cellulose fibers treated with different pre-gelation durations.

The SEM images of the cellulose fiber surfaces are shown in Figure 8. The diameter of regenerated cellulose fibers treated with different pre-gelation time is about 30 μm. Both the fibers without pre-gelation and those with pre-gelation lack the serrated outer surface characteristic of viscose fibers. The cross-sectional views of the fibers reveal that the shape of some fibers without pre-gelation is relatively irregular, whereas all pre-gelation fibers are nearly round (Figure S4 in ESI). The cross-sections of the cellulose fibers after tensile testing were also examined by SEM. The images in Figure 8 show that the cross-sections of the cellulose fibers remain circular and do not exhibit necking behavior.

**Figure 9:**
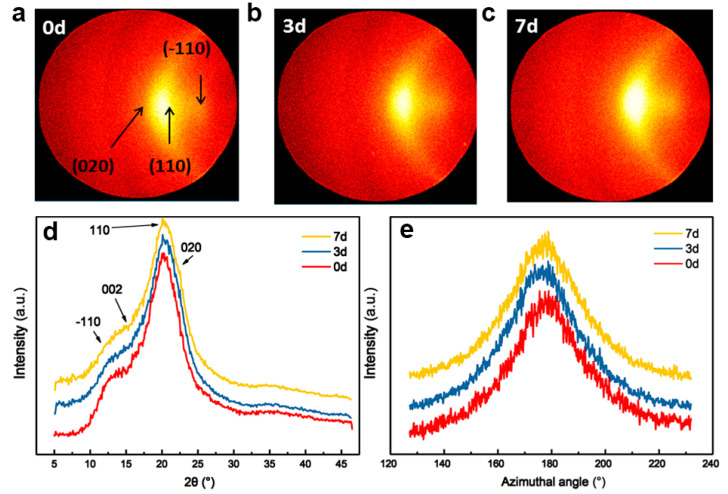
WAXD patterns of (**a**) G0, (**b**) G3, and (**c**) G7 fibers, (**d**) radial scanning curves, and (**e**) azimuthal scans of (110) plane.

Figure 9 summarizes the 2D WAXD patterns for cellulose fibers. There are two patterns that appear at 20.6° and 21.6°, corresponding to the characteristics of the cellulose II structure. The Azimuthal scans of regenerated fibers are plotted in Figure 9e. The (110) plane was selected for orientation analysis because its diffraction peak exhibits higher intensity and better resolution, while the (020) plane is weaker and overlaps with the (110) plane [31,32]. The FWHMs of the (110) plane azimuthal scans of the regenerated G0, G3, and G7 fibers are 32°, 29°, and 28°, respectively; the corresponding Herman’s orientation factors (*f*) are 0.70, 0.72, and 0.73, respectively. We can observe that the fiber orientation in pre-gelation samples is better than in those without pre-gelation. Also, the crystallinity degrees of G0, G3, and G7 fibers are 55%, 59%, and 59%, respectively, indicating that the crystallinity of regenerated cellulose is also somewhat improved by pre-gelation.

Based on the above results, pre-gelation of a cellulose solution represents a critical step that profoundly influences the crystallinity, molecular orientation, and tensile properties of regenerated cellulose materials. By initiating gelation under controlled conditions, such as temperature and time, cellulose chains are allowed to form entanglements and networks, facilitating better alignment and organization after fiber spinning. This pre-gelation phase promotes a reduction in the amorphous regions within cellulose structures, leading to enhanced crystallinity during subsequent regeneration processes. The controlled nucleation and growth of cellulose crystallites further contribute to the development of larger, more uniform crystalline domains. Consequently, the improved molecular alignment and crystallinity translate into superior tensile properties, including increased tensile strength and modulus, as observed in cellulose fibers or hydrogels. Moreover, pre-gelation ensures structural consistency by minimizing defects and irregularities, thereby enhancing the overall durability and performance of cellulose-based materials across diverse applications, from textiles to advanced biomedical products.

## 4. Conclusions

In conclusion, the study of cellulose dissolution, pre-gelation, and regeneration processes highlights significant advancements in understanding and optimizing the properties of regenerated cellulose materials. The use of pre-gelation step, particularly in solvent systems like LiCl/DMAc, has demonstrated its efficacy in enhancing the crystallinity, molecular orientation, and mechanical properties of regenerated cellulose fibers and hydrogels. The crystallinity of the fibers, as determined by X-ray diffraction, shows consistent improvement from 55% in untreated fibers (G0) to 59% in fibers pre-gelled for 3 and 7 days (G3 and G7). This indicates that pre-gelation promotes a more ordered arrangement of cellulose chains during regeneration. Moreover, pre-gelled fibers exhibit reduced full width at half of the maximum (FWHM) values in X-ray diffraction patterns, suggesting enhanced molecular alignment along the fiber axis. Mechanically, pre-gelled fibers demonstrate a 30% increase in tensile strength and a doubling of compression modulus compared to fibers without pre-gelation, highlighting their superior structural integrity and robustness. Overall, through careful control of gelation parameters such as temperature and duration, cellulose chains can form stable networks and crystalline structures, leading to improved tensile strength, modulus, and structural integrity. These findings reveal the pivotal role of pre-gelation in optimizing cellulose material properties, paving the way for applications in advanced textiles, biomaterials, and sustainable packaging where enhanced mechanical and structural characteristics are paramount. Continued research in this area promises further advancements in cellulose processing and utilization in high-performance materials.

## Data Availability

The authors confirm that the data supporting the findings of this study are available within the article.

## Supplementary Materials

The following supporting information can be downloaded at: https://www.mdpi.com/article/10.3390/ma17194886/s1, Figure S1: Typical fitting results of regenerated cellulose fiber XRD curve; Figure S2: WAXD curves of regenerated cellulose after dissolution in LiCl/DMAc at 5 °C and 25 °C; Figure S3: Angular frequency dependent of G′ (storage modulus) and G″ (loss modulus) of Cellulose/LiCl/DMAc solutions dissolved at 5 °C and 25 °C; Figure S4: Images of the cross section of regenerated cellulose fiber; Table S1: Young’s modulus, Tensile strength, strain, Crystallinity Index (CI), Full Width at Half Maximum (FWHM), Orientation Function (*f*).
