# Supplementary material for: Enhancing the Mechanical Properties of Regenerated Cellulose through High-Temperature Pre-Gelation
Source: Materials (Basel). 2024 Oct 5;17(19):4886. doi: 10.3390/ma17194886 (PMC11477954; doi:10.3390/ma17194886)
Supplement: Supplementary file 1 [file materials-17-04886-s001.zip › materials-3182466-supplementary.pdf]

Enhancing the Mechanical Properties of Regenerated Cellulose through High Temperature Pre-gelation

Yuxiu Yu[1,2], Weiku Wang[1,3] and Yaodong Liu[1,2,*]

[1] CAS Key Laboratory of Carbon Materials, Institute of Coal Chemistry, Chinese Academy of Sciences, 27 Taoyuan South Road, Taiyuan, 030001, China; yuyuxiu@sxicc.ac.cn (Y. Y.); weikuu7039@163.com (W. W.)
[2] Center of Materials Science and Optoelectronics Engineering, University of Chinese Academy of Sciences, Beijing 100049, China
[3] University of Chinese Academy of Sciences, 19 Yuquan Road, Beijing 100049, China
* Correspondence: liuyd@sxicc.ac.cn (Y. L.)

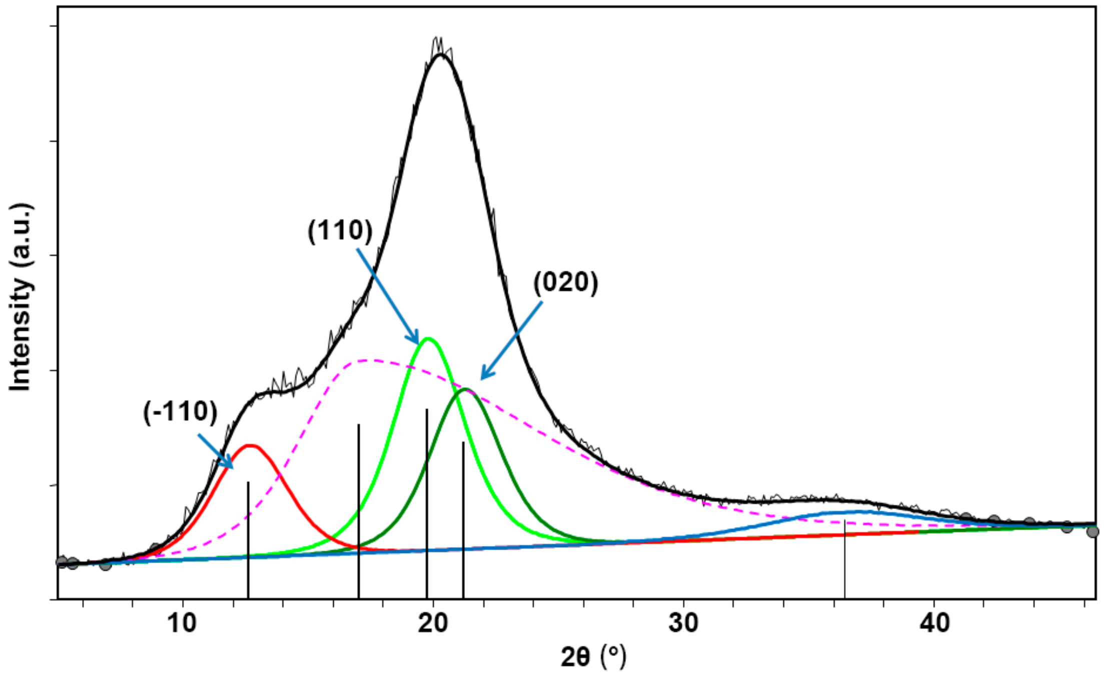

Figure S1. Typical fitting results of regenerated cellulose fiber XRD curve.

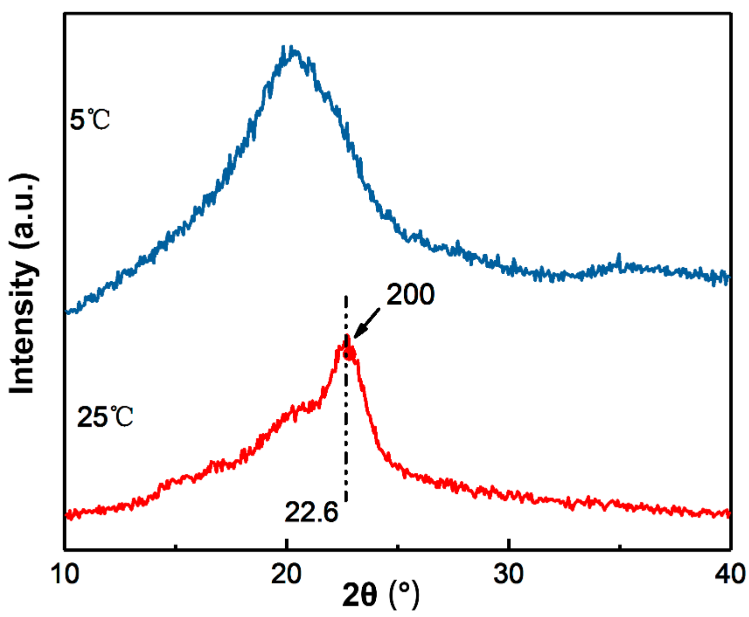

Figure S2. WAXD curves of regenerated cellulose after dissolution in LiCl/DMAc at 5°C and 25°C.

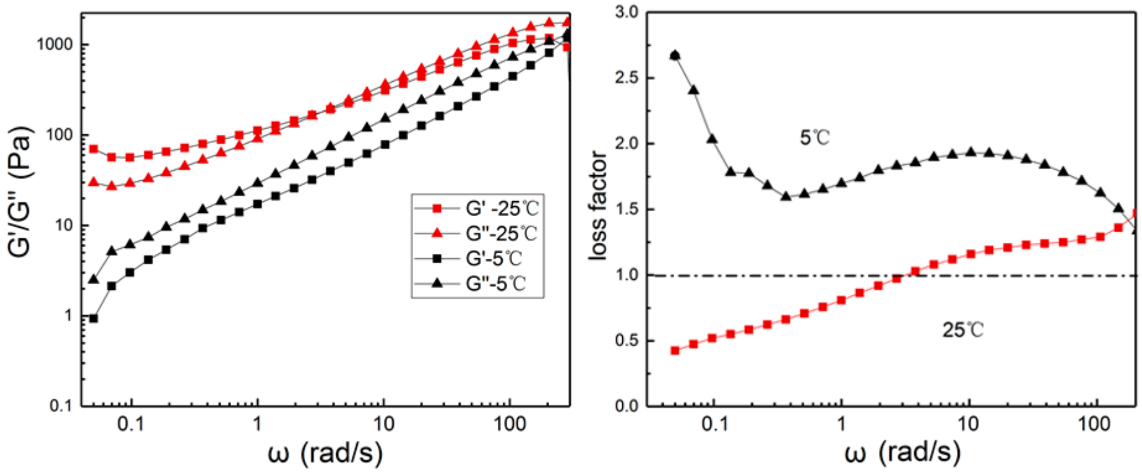

Figure S3. Angular frequency dependent of G'(storage modulus) and G'' (loss modulus) of Cellulose/LiCl/DMAc solutions dissolved at 5°C and 25°C.

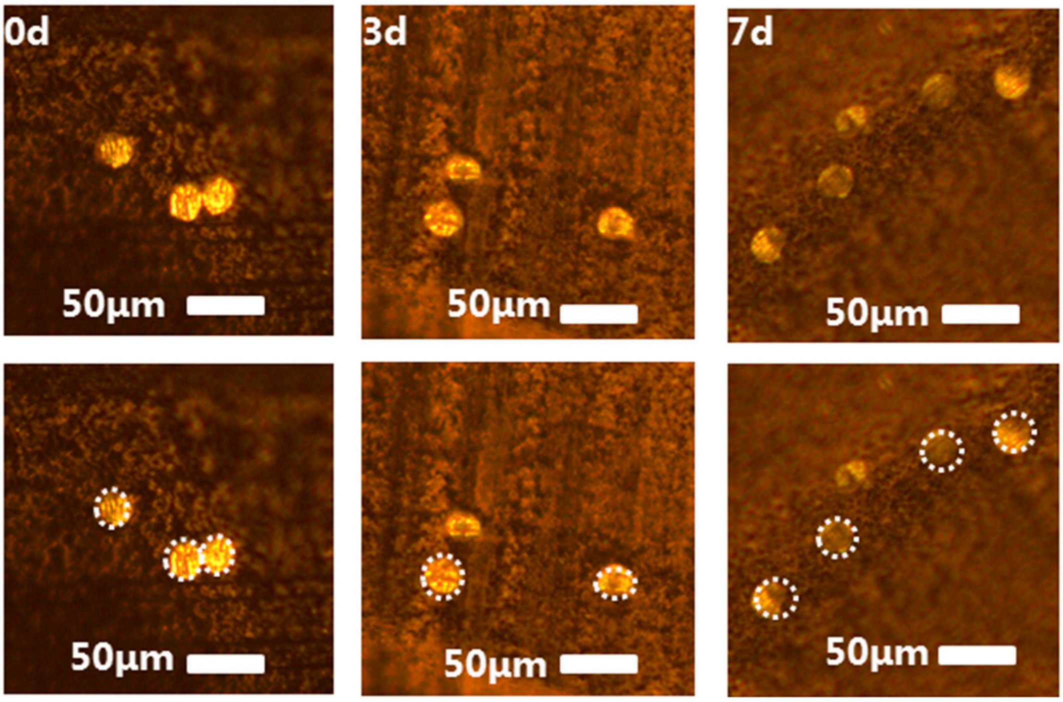

Figure S4. Images of the cross section of regenerated cellulose fiber.

Table S1. Young's modulus, Tensile strength, strain, Crystallinity Index (CI), Full Width at Half Maximum (FWHM), Orientation Function(*f*)

	**Young’s modulus** （**cN/dtex**）	**Tensile strength** （**cN/dtex**）	**Strain** （**%**）	**CI**（**%**）	**FWHM**	$f_{(110)}$
0d	101.0±5.4	1.52±0.08	7.0±0.9	55	31	0.70
3d	128.1±6.3	1.88±0.09	6.1±0.9	59	29	0.72
7d	139.3±4.2	2.20±0.08	5.6±1.1	59	28	0.73
